# Acne Vulgaris and adherence to the mediterranean diet among university students: a case‒control study

**DOI:** 10.1186/s41043-024-00535-1

**Published:** 2024-03-13

**Authors:** Sari Taha, Muna Shakhshir, Sa’ed H. Zyoud

**Affiliations:** 1 An-Najah Global Health Institute, An-Najah National University, Nablus, 44839 Palestine; 2 Department of Anatomy, Biochemistry and Genetics, An-Najah National University, Nablus, 44839 Palestine; 3 Department of Nutrition, An-Najah National University Hospital, Nablus, 44839 Palestine; 4 Department of Clinical and Community Pharmacy, College of Medicine and Health Sciences, An-Najah National University, Nablus, 44839 Palestine; 5 Poison Control and Drug Information Center (PCDIC), College of Medicine and Health Sciences, An-Najah National University, Nablus, 44839 Palestine; 6 Clinical Research Center, An-Najah National University Hospital, Nablus, 44839 Palestine

**Keywords:** Acne Vulgaris, Mediterranean diet, Acne severity, Dermatology, Nutrition, Acne

## Abstract

**Background:**

Western diets, characterized by a high glycemic index and dairy content, can be risk factors for acne vulgaris. A few studies have suggested that adherence to non-Western diets, such as the Mediterranean diet (MD), may be protective against acne. This study aimed to explore the relationships between adherence to the MD and acne diagnosis and severity.

**Methods:**

This was a matched case‒control study carried out among university students studying health sciences to explore the relationship between adhering to the MD and an acne diagnosis. Convenience sampling was utilized for the initial recruitment of eligible participants, who were then 1:1 individually matched for age, gender, and body mass index (BMI). Adherence to the MD was assessed using the Mediterranean Diet Adherence Screener (MEDAS) tool, and acne severity was assessed using the Global Acne Grading System (GAGS). The data were analyzed using descriptive statistics, bivariate analysis, and conditional logistic regression, which included two models based on clinical data and the backward elimination technique.

**Results:**

A final sample of 121 cases was individually matched to 121 controls. Each group consisted of 28.9% males and 71.1% females, with most having a BMI within the healthy range (71.9%). Both the case (80.2%) and control groups (77.7%) demonstrated a predominant pattern of low adherence to the MD. At the bivariate level, family history significantly differed between the case and control groups (OR = 2.01, CI = 1.17–3.44), while adherence to the MD (OR = 0.86, CI = 0.46–1.60) did not reach statistical significance. According to the regression analysis, family history remained significant in the backward elimination model (aOR = 1.94, CI = 1.14–3.34), while it disappeared in the full model (aOR = 1.95, CI = 1.14–3.34). Neither model revealed a significant association between acne and the other variables. Among the participants in the case group, neither adherence to the MD nor adherence to its components was significantly associated with acne severity, except for vegetables (*p* = 0.022).

**Conclusions:**

Adherence to the MD was not correlated with acne diagnosis or clinical severity. More research on the association between acne and adherence to the MD is needed, as earlier studies are few, were conducted in specific settings, and used variable methodologies. To improve the validity and reliability of the research methodology, the development of detailed and culturally adapted MD definitions and practical guides is recommended.

## Introduction

Acne vulgaris is a common dermatological disease with a chronic, inflammatory course. While the global prevalence of acne vulgaris was estimated to be 3,073.3 cases per 100,000 people in 2019 according to the Global Burden of Disease (GBD) study, the prevalence among adolescents and young adults is higher and substantially decreases beyond 30 years of age [1–3]. The regional prevalence is higher in high-income regions, such as Western Europe; however, the change in acne prevalence between 1990 and 2019 was among the greatest in the Eastern Mediterranean Region (EMR) [2, 3]. The reported prevalence of acne in the region ranges widely from 34.7% in Syria to 80.9% in Palestine, with a general trend of female predominance [4–7]. Moreover, acne is more prevalent and severe among males in late adolescence [8, 9] but more prevalent in females across other age groups [10, 11].

Several mechanisms are implicated in the multifaceted and interdependent pathogenesis of acne, including inflammatory mechanisms, excessive sebum production, keratinocyte hyperplasia, androgenic hormone effects, and hyperproliferation of *cutibacterium acnes* [12, 13]. Insulin-like growth factor-1 (IGF-1) induces hyperplasia of keratinocytes and increases sebum production by promoting sebaceous gland hyperproliferation and lipid synthesis [12, 14, 15]. Given the impact of diet on the hormonal, inflammatory, and environmental factors leading to acne, various dietary components have been found to affect acne development. Irrespective of culinary characteristics, a high glycemic index (GI) diet has been linked to acnegenesis, whereas adoption of a low-GI diet is associated with reduced acnegenesis and severity in adolescents and young adults [16–20]. However, evidence on milk consumption is mixed and largely dependent on observational studies. Increased dairy intake is associated with acne severity in young populations only in areas where Western diets prevail [21–23]. Both high-GI and dairy diets lead to hyperinsulinemia and elevated IGF-1 levels, thereby increasing sebum and androgen hormone production, both of which are implicated in acne pathophysiology [15, 24, 25]. Furthermore, the consumption of omega-3 fatty acids and γ-linoleic acid, which are abundant in fish and olive oil, may improve acne symptoms [26].

The Mediterranean diet (MD) is mainly a plant-based diet that was developed and adopted around the Mediterranean Basin before globalization impacted traditional dietary systems. The MD is based on a regular intake of olive oil as the primary source of fat and high consumption of fruits, vegetables, and grains, while allowing a moderate intake of white meat, fish and wine, and restricting the consumption of sugary products and red meat [27, 28]. Research has suggested that adherence to the MD may reduce the risk of cardiovascular disease (CVD), type 2 diabetes mellitus, metabolic syndrome, dementia, rosacea, psoriasis, and hidradenitis suppurativa [29–35]. These health benefits have been attributed to the effects of the MD on insulin sensitivity, endothelial function, blood lipids, and inflammatory and oxidative stress [36].

The assumption that non-Western diets, such as the MD, may have a protective role against acne is based on two arguments. First, observational studies of non-Western populations have suggested a protective role of non-Western diets against acne. One study, for example, reported no cases of acne among two South American indigenous populations, while the introduction of Western diets and lifestyles increased the incidence of acne [37]. Second, the individual dietary components of the MD demonstrate biological plausibility for acne protection. The MD components have a low GI and contain antioxidant compounds with anti-inflammatory effects, such as hydroxytyrosol and tyrosol, which are present in olive oil [38].

Research on the impact of adherence to a whole dietary pattern, such as the MD, on acne is scarce. Only a few studies have suggested a protective effect of adherence to the MD on acne [39–42]. However, these studies were limited to populations residing in southern European countries. Palestine is an Eastern Mediterranean area with distinctive and diverse cuisine. Despite this diversity, Palestinian cuisine is based on common dietary characteristics, such as the high intake of olive oil, vegetables and white meat, which are basic elements of the MD [43, 44]. This case‒control study aimed to investigate the impact of the MD on the presentation and severity of acne among university health science students in Palestine. It also aimed to explore the extent of adherence to the MD and its components.

## Methods

### Study design and settings

This was a matched case‒control study that was conducted using clinical examination and an interviewer-administered questionnaire to assess adherence to the MD for one year preceding the study, as the exposure of interest, among the case and control groups, with acne vulgaris as the outcome of interest. It was performed among students of health sciences majors at An-Najah National University in Nablus, Palestine between March 15 and July 31, 2023.

### Sampling and selection of case and control groups

The case definition of acne is the presence of comedones, papules, pustules, nodules, and/or cysts on the face and upper trunk as identified by a clinical examination conducted by a certified physician under clear daylighting [45]. The cases were students diagnosed with acne as per the abovementioned case definition, and the controls were acne-free students to whom the case definition did not apply and who belonged to the same population base of students as the cases.

The case and control groups were individually matched for age, gender, and body mass index (BMI). Matching improves the statistical efficiency of an effect estimator by reducing the variance [46]. Matching variables were selected based on the presence of a potential association between the outcome and exposure, given that matching for a particular variable is practical and feasible. For age, the participants were matched according to three age groups: 18–19, 20–21, and ≥ 22 years old. For BMI, the participants were matched according to the World Health Organization’s (WHO) BMI classification: underweight, < 18.5 kg/m²; normal weight, 18.5–24.9 kg/m²; overweight, 25.0-29.9 kg/m²; and obese, > 30.0 kg/m² [47].

Female and male students enrolled at the Faculty of Medicine and Health Sciences and registered in the second or summer semester were eligible to participate. Students with a past medical history of diabetes, hypertension, polycystic ovarian syndrome (PCOS), hidradenitis suppurativa, and corticosteroid-induced acne and those previously treated with isotretinoin for one year before the study were excluded from the study. A convenience sampling technique was used by inviting the students to participate in the study. Invitations were communicated during lectures given at the Faculty of Medicine and Health Sciences. The following equation was used to determine the sample size [48, 49]:

n (for each group) = (*r* + 1) (p*) (1-p*) (Z [α/_2]_ + Z [β])^2^/r (p_0_-p_1_)^2^.

Z [α/_2]_ value corresponding to a significance level of 0.05 = 1.96.

Z [β] value corresponding to a desired power of 0.20 = 0.84.

p*= p_0+_p_1_/2.

r = ratio of cases to controls.

*p*_*0*_ is the proportion of controls with exposure, which was assumed to be 0.625 based on a previous study on adherence to the MD among a similar population of Palestinians [50].

*p*_*1*_ is the proportion of cases with exposure. *p*_*1*_ was estimated at 0.431 based on data from the only study investigating the impact of adherence to the MD and acne, using the same questionnaire, and reporting the proportion of cases with exposure [40].

The sample size yielded by the equation was 106 for each group. To increase the power of the study, the final sample size was increased depending on the ability to recruit, include and match eligible cases and controls. Students who were eligible for inclusion were examined for acne and joined the potential pool of the case and control groups. Then, candidates were checked for matching based on information obtained on the matching variables. Cases and controls who could not be matched to an appropriate candidate from the other group were excluded. When more than one case or control could be matched to only one candidate, the case‒control pair was selected randomly using computer-generated numbers. All successfully matched pairs were enrolled in the study.

### Data collection

#### I. definitions of demographic, personal and clinical variables

The interviewer-administered questionnaire included demographic, personal, and clinical questions on age, gender, major, year of study, self-reported weight and height, smoking status, consumption of milk, duration of acne (categorized as < 1 year, 1–5 years, 5 to 10 years, > 10 years), family history of acne, and self-reported, past medical history of diabetes mellitus, hypertension, metabolic syndrome, dyslipidemia, polycystic ovarian syndrome (PCOS), and use of steroids or isotretinoin during the year preceding the study. Smoking status was classified into three categories: current smoker, defined as a participant who smoked cigarettes, vape or hookah on most days of the past year; previous smoker, defined as a participant who quit smoking more than one year before the start of the study; and nonsmoker, defined as a participant to whom the previous two conditions did not apply. Milk consumption was classified into frequent consumption on most days, once to twice a week, or less than once a week. The questionnaire included questions on milk consumption, smoking status, and family history as confounders to be adjusted for in the analysis [51].

#### II. Dietary assessment

The dietary assessment was made using the interviewer-based 14-Item Mediterranean Diet Adherence Screener (MEDAS) tool to retrospectively assess adherence to the MD during the year preceding the study [52–55]. The MEDAS tool was developed for use in the PREDIMED study, a large randomized controlled trial investigating the role of adherence to the MD in the primary prevention of CVD [56]. Given its short and simple content, the MEDAS tool provides quick and practical estimation of adherence to the MD and requires less effort than longer screening tools while maintaining validity and reliability. Indeed, the tool has demonstrated validity and reliability in different countries and languages [52, 57–59]. Moreover, the content and arrangement of the questionnaire were found to be culturally appropriate for the population targeted in this study. Furthermore, previous studies exploring the association between adherence to the MD and acne use the same tool for assessment, which might facilitate comparison to these studies [40, 41]. The MEDAS questionnaire comprised 12 inquiries regarding the frequency of food consumption and two queries related to dietary habits. A maximum score of 14 can be attained by higher consumption frequency of eight food components, the adoption of two dietary habits, and lower consumption frequency of four components. One point is assigned for each of the following criteria: reliance on olive oil as the primary source of fat, preference for white meat over red meat, intake of olive oil (≥ 4 tablespoons/day), inclusion of vegetables (≥ 2 servings/day), incorporation of fruits (≥ 3 servings/day), limited consumption of red or processed meats (< 1 serving/day), restricted use of butter, cream, or margarine (< 1 serving/day), minimal intake of sweet or carbonated beverages (< 1 cup/day, cup = 100 ml), inclusion of legumes (≥ 3 servings/week), regular consumption of fish or seafood (≥ 3 servings/week), limited intake of commercial sweets and pastries (< 2 pieces/week), incorporation of tree nuts (≥ 3 servings/week), regular consumption of wine (≥ 7 glasses/week), and utilization of sofrito sauce (≥ 2 servings/week) [52]. Sofrito sauce, composed of garlic, onion, and tomatoes sautéed in olive oil, is the basis for this assessment [60]. Participants are categorized based on their final scores, with a score of ≤ 7 indicating low adherence to the Mediterranean diet and a score > 7 denoting higher adherence to the diet. Professor Miguel A Martínez-González granted permission to use the scale via email.

#### III. Assessment of acne severity

The Global Acne Grading System (GAGS) was used to assess acne severity. The GAGS assigns a severity score based on the location and form of the acne lesions. For locations, 3 points are given for lesions on the trunk; 2 for those on each of the forehead, right cheek, and left cheek, and 1 for each of those on the nose or chin. For forms, only the most severe lesion was considered for grading, with 1 point given for comedones, 2 for papules, 3 for pustules, and 4 for nodules and/or cysts. To calculate the local score, the number of points given for a particular location is then multiplied by the number of points for the most severe lesion on that location. The global score is the summation of the local grade of every involved location. The severity of acne was graded according to the global score as follows: mild (GAGS: 1–18), moderate (GAGS: 19–30), severe (GAGS: 31–38), or very severe (GAGS > 38) [61]. A binary outcome of mild (≤ 18) and moderate-to-severe (> 18) acne was used in this study.

### Validity and reliability

A set of measures was taken to enhance the validity and reliability of the study. First, cases were defined based on a clear reference to avoid classification error, and controls were selected independent of exposure status and from the same base population that produced the cases. Moreover, confounding factors were controlled by matching, restriction, and regression analysis. Restriction was performed by excluding cases with diseases and medications that may affect the relationship between diet and acne. Finally, matched analysis using the appropriate method of regression was conducted to account for potential confounders.

### Data analysis

Statistical analysis was performed using SPSS version 26.0 (IBM Corporation, USA). The Shapiro‒Wilk test was used to test for normality of the MD score. The case and control groups were compared for differences in demographic and lifestyle characteristics and adherence to the components of the MD using frequency and percentage. The mean and standard deviation were reported for age. Bivariate analysis to assess differences in exposure employed a matched analysis using McNemar’s chi-square test [62]. The crude odds ratio of potential confounding exposures among the participants in the case and control groups was calculated. As the study adopted an individual 1:1 matching approach, two conditional logistic regression models were developed and used to adjust the odds ratio to potential confounders. Conditional logistic regression, which ‘*conditions*’ each case to its matched control, is used in individually matched case‒control studies to avoid the inaccuracy that might arise from potential small values in the strata of contingency Table [62]. The full regression model included potential confounders by *a priori* clinical selection of variables based on the literature, while the other model employed the backward elimination technique to select the variables that would be included in the most parsimonious model fitting the data [63, 64]. The goodness of fit for the second model was assessed using the Hosmer and Lemeshow test (a *p* value > 0.05 indicates a good fit). Furthermore, the chi-square test was used to analyze the association between acne severity and adherence to the MD components among the participants in the case group. The precision of association was determined using a 95% confidence interval (CI) and a *p* value < 0.05.

### Ethical consideration

Permission to conduct this study was obtained from the Institutional Review Board (IRB) of An-Najah National University. The study was conducted in accordance with the relevant regulations of the Helsinki Declaration [65]. Informed consent was obtained from the participants, with privacy and confidentiality ensured during the clinical examination.

## Results

**Fig. 1:**
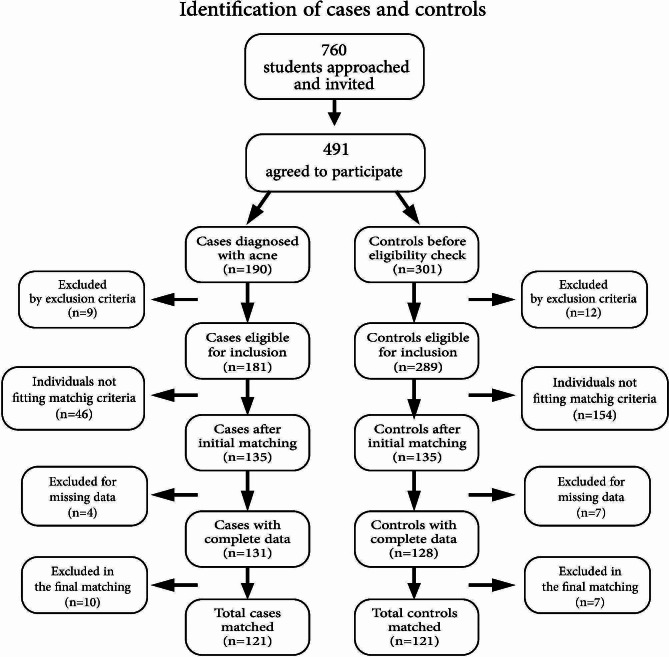
The process of recruitment, selection and matching of the case and control groups

Of the 5200 registered students potentially eligible for inclusion, 760 were approached and invited to participate. Of those, 491 agreed to participate and consented to the examination, for a study response rate of 64.6%. After examination, 190 cases were diagnosed with acne, nine of whom were excluded according to the exclusion criteria. The potential control group consisted of 301 participants, 12 of whom were excluded, resulting in the matching of 289 potential control candidates with 181 cases. Matching yielded two groups of 135 cases and 135 controls who completed the questionnaire. Then, four cases and seven controls were excluded due to considerable missing data, resulting in a survey response rate of 97.0% among cases and 94.8% among controls. To achieve optimal matching, a further ten cases and seven controls were excluded. The final sample consisted of 121 individually matched cases and 121 controls. Due to the nature of recruitment, reasons for nonparticipation other than noneligibility and missing data could not be identified. Figure 1 illustrates the process of recruitment, selection and matching.

The mean ages of the participants in the case and control groups were 20.3 (*SD* ± 1.7) and 20.1 (*SD* ± 2.3), respectively. Each group consisted of 28.9% (*n* = 35) males and 71.1% (*n* = 86) females. Most participants in both groups had a BMI within the healthy range (71.9%, *n* = 174). Medicine, dentistry and nursing were the most common study majors reported by the participants in both the case and control groups. The majority of the participants in the case (72.7%, *n* = 88) and control (57.0%, *n* = 69) groups reported a positive family history of acne. While most participants in the case (85.5%, *n* = 100) and control (89.0%, *n* = 97) groups said they were nonsmokers, just less than half of the participants in the case (42.4%, *n* = 50) and control (44.4%, *n* = 48) groups reported daily intake of milk (Table 1).

**Table 1:** Background characteristics of the case and control groups

Factors	Cases N (%)	Controls N (%)	Total	p value
**Age**				-
18–19	42 (34.7)	42 (34.7)	84 (34.7)	
19–20	50 (41.3)	50 (41.3)	100 (41.3)	
≥21	29 (24.0)	29 (24.0)	58 (24.0)	
**Gender**				-
Male	35 (28.9)	35 (28.9)	70 (28.9)	
Female	86 (71.1)	86 (71.1)	172 (71.1)	
**BMI**				-
Underweight	10 (8.3)	10 (8.3)	20 (8.3)	
Healthy range	87 (71.9)	87 (71.9)	174 (71.9)	
Overweight	21 (17.4)	21 (17.4)	42 (17.4)	
Obese	3 (2.5)	3 (2.5)	6 (2.5)	
**Study Major**				0.197
Medicine	64 (52.9)	77 (63.6)	141 (58.3)	
Dentistry	8 (6.6)	14 (11.6)	22 (9.1)	
Nursing	12 (9.9)	7 (5.8)	19 (7.9)	
Pharmacy	7 (5.8)	5 (4.1)	12 (5.0)	
Medical imaging	8 (6.6)	2 (1.7)	10 (4.1)	
Speech pathology	2 (1.7)	5 (4.1)	7 (2.9)	
Anesthesia and Resuscitation	5 (4.1)	2 (1.7)	7 (2.9)	
Pharmacy doctor	3 (2.5)	2 (1.7)	5 (2.1)	
Physiotherapy	3 (2.5)	2 (1.7)	5 (2.1)	
Optometry	3 (2.5)	1 (0.8)	4 (1.7)	
Cardiac perfusion technology	1 (0.8)	2 (1.7)	3 (1.2)	
Medical Laboratory Sciences	3 (2.5)	0 (0.0)	3 (1.2)	
Midwifery	2 (1.7)	1 (0.8)	3 (1.2)	
Cosmetics and skin Care	1 (0.8)	0 (0.0)	1 (0.4)	
**Year of study**				0.643
First year	38 (31.4)	28 (23.1)	66 (27.3)	
Second year	36 (29.8)	42 (34.7)	78 (32.2)	
Third year	22 (18.2)	22 (18.2)	44 (18.2)	
Fourth year	15 (12.4)	17 (14.0)	32 (13.2)	
Fifth year	9 (7.4)	12 (9.9)	21 (8.7)	
Sixth year	1 (0.8)	0 (0.0)	1 (0.4)	
**Family history of acne vulgaris**				
Positive	88 (72.7)	69 (57.0)	157 (64.9)	0.011*
Negative	33 (27.3)	52 (42.9)	58 (35.1)	
**Adherence to the MD**				
Low	97 (80.2)	94 (77.7)	191 (78.9)	0.652
High	24 (19.8)	27 (22.3)	51 (21.1)	
**Milk Consumption**				0.736
None	28 (23.7)	21 (19.4)	49 (21.7)	
1–2 per week	40 (33.9)	39 (36.1)	79 (35.0)	
Daily intake	50 (42.4)	48 (44.4)	98 (43.4)	
**Smoking status**				0.700
Nonsmoker	100 (85.5)	97 (89.0)	197 (87.2)	
Previous smoker	2 (1.7)	1 (0.9)	3 (1.3)	
Current smoker	15 (12.8)	11 (10.1)	26 (11.5)	

*: *p* value is below the threshold for significance (0.05). McNemar’s chi squared test was used to test for statistical significance

- Abbreviations: BMI: body mass index, MD: Mediterranean diet

The MD score was found to be nonnormally distributed (Shapiro–Wilk test, *p* < 0.001). The majority of both the case (80.2%, *n* = 97) and control groups (77.7%, *n* = 94) had low adherence to the MD, with the same median score of 6.0 (IQR = 5.0–7.0). Of the 14 survey items, only six indicating high adherence were predominant: utilization of oil as the main fat source (74.8%, *n* = 181) coupled with low consumption of animal fat sources (12.0%, *n* = 29); high consumption of dishes with sofrito sauce (78.1%, *n* = 189); consumption of poultry more than red meat (57.9%, *n* = 140) coupled with low consumption of red or processed meat (37.6%, *n* = 91); and low consumption of sweet or carbonated beverages (25.6%, *n* = 62) (Table 2).

**Table 2:** Description of adherence to the components of the MD (MEDAS questionnaire) among the acne and control groups

Item	Cases N (%)	Controls N (%)	Total N (%)	p value
Use of olive oil as main source of fat	90 (74.4)	91 (75.2)	181 (74.8)	0.882
olive oil ≥ 4 tablespoons/day	38 (31.4)	39 (32.2)	77 (31.8)	0.890
Vegetables ≥ 2 servings/day	26 (21.5)	42 (34.7)	68 (28.1)	0.022*
Fruits ≥ 3 servings/day	18 (14.9)	20 (16.5)	38 (15.7)	0.724
Red or processed meats ≥ 1 serving/day	46 (38.0)	45 (37.2)	91 (37.6)	0.894
Butter, cream, or margarine ≥ 1 serving/day	17 (14.0)	12 (9.9)	29 (12.0)	0.322
Sweet or carbonated beverages ≥ 1 cup/day	34 (28.1)	28 (23.1)	62 (25.6)	0.377
Legumes ≥ 3 servings/week	38 (31.4)	33 (27.3)	71 (29.3)	0.480
Fish and seafood ≥ 3 servings/week	11 (9.1)	9 (7.4)	20 (8.3)	0.641
Commercial sweets ≤ 2/week	78 (64.5)	70 (57.9)	148 (61.2)	0.291
Tree nuts ≥ 3 servings/week	33 (27.3)	39 (32.2)	72 (29.8)	0.399
Poultry more than red meats	75 (62.0)	65 (53.7)	140 (57.9)	0.193
Use of sofrito sauce in a dish ≥ 2 servings/week	90 (74.4)	99 (81.8)	189 (78.1)	0.162

*: *p* value is below the threshold for significance (0.05). The chi-squared (χ²) test was used to test for statistical significance

- The frequency and percentage values represent the number of participants in the case and control groups with higher consumption in relation to the total number of participants in the case and control groups, respectively. The frequency and percentage values under the column “total” represent the total number of participants with higher consumption in relation to the number of participants

- A higher consumption of red/processed meat; butter, cream, or migraine; sweet or carbonated beverages; and commercial sweets indicates low adherence to the MD.

At the bivariate level, only having a positive family history of acne was significantly different between the case and control groups (*p* = 0.011) (Table 1). The proportion of participants with high adherence to the MD in the control group (*n* = 27, 22.3%) was higher than that in the case group (*n* = 24, 19.8%). Likewise, the control group demonstrated a trend toward higher adherence to the individual components of the MD. However, these case‒control differences in adherence to either the MD (*p* = 0.652) or to the individual MD components did not reach statistical significance, except for vegetable consumption (*p* = 0.022) (Table 2). The conditional logistic regression based on backward elimination selected family history and adherence to the MD as the variables of the best model fitting the data *(Hosmer and Lemeshow test, p =* 0.343*).* Similar to the bivariate analysis, acne cases were significantly more likely to have a positive family history (aOR = 1.94, CI = 1.14–3.34), while adherence to the MD did not achieve statistical significance (aOR = 0.88, CI = 0.47–1.62). The full regression model showed that none of the variables, including family history, were associated with acne (Table 3).

**Table 3:** Conditional logistic regression of the variables potentially affecting acne diagnosis

	Crude odds ration	Full model: adjusted odds ratio (CI 95%)	Backward elimination model: adjusted odds ratio (CI 95%)
**Adherence to the MD**			
Low adherence	reference	reference	reference
High adherence	0.86 (0.46–1.60)	0.70 (0.34–1.37)	0.88 (0.47–1.62)
**Family history of acne vulgaris**			
Negative family history	Reference	reference	reference
Positive family history	2.01* (1.17–3.44)	1.75 (0.91–3.13)	1.95* (1.14–3.34)
**Smoking status**			
Nonsmoker	reference	reference	-
Previous smoker	2.19 (0.19–24.83)	1.51 (0.12–18.64)	
Current smoker	1.31 (0.51–3.35)	1.19 (0.45–3.17)	
**Milk Consumption**			
None	reference	reference	-
1–2 times a week	1.10 (0.56–2.17)	0.83 (0.42-2.00)	
Daily intake	1.01 (0.54–1.85)	0.72 (0.41–1.83)	

- Abbreviations: CI: Confidence interval, MD: Mediterranean diet

- Analysis was conducted by employing conditional logistic regression. The full regression model included potential confounders by clinical selection of variables based on the literature (family history of acne vulgaris, smoking status, and milk consumption). The second model used the backward elimination technique, and adherence to the MD and family history of acne vulgaris were the only two variables that fit the model

- The Hosmer–Lemeshow test indicated a goodness of fit (*p* value > 0.05) for both models

Most of the participants in the case group had acne on the forehead (65.3%, *n* = 79), followed by the right cheek (55.4%, *n* = 76) and left cheek (52.1%, *n* = 63). Nearly one-third had acne for less than one year (33.9%, *n* = 41), while almost half had acne for one to five years (51.2%, *n* = 62) (Table 4). Among the participants in the case group, adherence to the MD was not significantly associated with acne severity (*p* = 0.579). In addition, none of the MD components were significantly associated with acne severity (see Table 5).

**Table 4:** Clinical characteristics of the case group

Clinical characteristics	Frequency (%)
**Location**	
Forehead	79 (65.3)
Right cheek	76 (55.4)
Left cheek	63 (52.1)
Nose	18 (14.9)
Chin	51 (42.1)
Trunk	32 (26.4)
**Duration of acne**	
Less than one year	41 (33.9)
One to five years	62 (51.2)
Five to ten years	17 (14.0)
More than ten years	1 (0.8)
**Family history of acne**	
Positive family history	88 (72.7)
Paternal family history	12 (9.9)
Sibling family history	76 (62.8)
Negative family history	33 (27.3)

**Table 5:** Relationships between the Mediterranean diet components and acne severity among participants in the case group

Factors	Mild acne Frequency (%)	Moderate-to-severe acne Frequency (%)	p value
**Adherence to the MD**			0.579
Low	74 (81.3)	23 (76.7)	
High	17 (18.7)	07 (23.3)	
**Use of olive oil as main source of fat olive oil**			0.880
No	23 (25.3)	8 (6.6)	
Yes	68 (74.7)	22 (73.3)	
**Consumption of olive oil**			0.793
<4 tablespoons/day	63 (69.2)	20 (66.7)	
≥4 tablespoons/day	28 (30.8)	10 (33.3)	
**Vegetables**			0.459
<2 servings/day	70 (76.9)	25 (83.3)	
≥2 servings/day	21 (23.1)	5 (19.2)	
**Fruits**			0.236
<3 servings/day	75 (82.4)	28 (93.3)	
≥3 servings/day	16 (17.6)	2 (6.7)	
**Red or processed meats**			0.542
< 1 serving/day	55 (60.4)	20 (66.7)	
≥1 serving/day	36 (39.6)	10 (33.3)	
**Butter, cream, or margarine**			0.362
<1 serving/day	80 (87.9)	24 (80.0)	
≥1 serving/day	11 (12.1)	6 (20.0)	
**Sweet or carbonated beverages**			0.789
< 1 drink/day	66 (72.5)	21 (70.0)	
≥1 drink/day	25 (27.5)	9 (30.0)	
**Legumes**			0.279
< 3 servings/week	60 (65.9)	23 (76.7)	
≥3 servings/week	31 (34.1)	7 (23.3)	
**Fish and seafood**			
< 3 servings/week	82 (90.1)	28 (93.3)	0.730
≥3 servings/week	9 (9.9)	2 (6.7%)	
**Commercial sweets**			0.882
<2/week	32 (35.2)	11 (36.7)	
≥2/week	59 (64.8)	19 (63.3)	
**Tree nuts**			0.576
<3 servings/week	65 (71.4)	23 (76.7)	
≥3 servings/week	26 (28.6)	7 (23.3)	
**Poultry consumption more than red meats**			0.542
No	36 (39.6)	10 (33.3)	
Yes	55 (60.4)	20 (66.7)	
**Use of sofrito sauce in a dish**			0.195
< 2 servings/week	26 (28.6)	5 (16.7)	
≥ 2 servings/week	65 (71.4)	25 (83.3)	

*: *p* value is below the threshold for significance (0.05)

- The chi-squared (χ²) test was used to test for statistical significance

- Abbreviations: MD: Mediterranean diet

## Discussion

The impact of diet on acne pathogenesis is controversial. Particularly, high GI and dairy intake, both of which are characteristic of Western diets, are associated with acnegenesis. The MD, as a non-Western diet consisting of foods with high anti-inflammatory and low-GI components, may reduce the odds of acne. However, only a few recent studies have explored the impact of adherence to the MD on acne. This case‒control study aimed to investigate the impact of adherence to the MD on acne and assess the extent of this adherence in a young population of university students.

The study revealed that adherence to the MD is not associated with acne diagnosis or severity. In contrast, three previous studies adopting a case‒control design have suggested that high adherence to the MD may be protective against acne [39–41]. This contrast may be attributed to variations in research settings and methodologies, thereby limiting comparisons to the present study. First, all of these studies were conducted in southern European countries, namely Italy and France, with one study restricted to middle-aged females. Cultural disparities in dietary patterns and lifestyles may contribute to differences in overall diet, which may confound the relationship between adherence to the MD and acne development, leading to varying effects in different settings. Furthermore, one study utilized a different scale with different dietary components and a distinct score calculation [39]. Another study reported an association between adherence to the MD and clinical severity among participants in the case group, but it did not report a difference between the case and control groups [41]. Given the paucity of related studies and their methodological variations, the generalization of a protective effect of adherence to the MD on acne amounts to hasty generalization, which is an informal fallacy of extrapolation based on insufficient evidence [66, 67]. Instead, the hypothesis and little evidence arising from these published studies need further longitudinal research to integrate the MD into a holistic, dietary approach for acne management.

This limitation in comparison is a part of the broader context of nutrition epidemiology where the validity of diet research is compromised by inherent methodological challenges in measurement and adjusting for confounding. By nature, diets and nutritional status are influenced by biological variations in nutrient absorption and metabolism and interact with a multitude of lifestyle factors, such as physical activity and smoking [68]. Comprehensive identification of potential confounders between acne and adherence to the MD is thus impractical and infeasible, especially since the association between these factors and acne remains highly inconclusive [51]. Moreover, diets consist of various nutritional components, making it challenging to isolate the impact of certain nutrients consumed within a whole diet [69].

Furthermore, accurate measurement of dietary exposure is challenging for several reasons. Because quantification of portion size, which varies across regions, is not specified in standardized scoring tools for the general population, the reliability of these tools is questionable [70]. The development of culturally appropriate nutritional guides may help adjust data collection tools for use in different settings. For example, a photographic food atlas containing portion size estimations for certain foods was created in Palestine [71], which can be further developed, systemized and published for use in research methodology conducted in the region. Moreover, as diet impact on health is accumulative, a longitudinal, prospective design is crucial for establishing a cause‒effect relationship based on a valid temporal sequence of diet and disease. Even when such a design is adopted, eating habits are inconsistent over time due to changing life conditions and seasonal variations [68, 69].

The MD definition is broad and nonstandardized, as it has been described based on general trends in food consumption [28, 70]. Instead, the conceptualization of the MD should move toward formulating comprehensive, detailed, and culturally suited definitions. The traditional definition of the MD, on which the scoring systems were based, overlooks the cultural nuances in the eastern and southern Mediterranean [70]. The different Mediterranean cuisines are heterogeneous, although they share the major elements of the MD by virtue of similarities in geography, culture, and climate. In these cuisines, the MD is a part of a broader dietary system, which may confound the accumulative effect of diet on acne. Noah and Truswell described the culinary variations across the Mediterranean and proposed a regional classification of countries into four groups based on these variations. Eastern cuisine, which includes Palestine, is characterized by high consumption of white flour, rice, eggs, and specific varieties of beans, herbs and cocked vegetables [72]. Exploring and describing local dietary systems is recommended to guide the development of culturally tailored nutrition surveys.

The present study revealed a low prevalence of high adherence to the MD among university students in Palestine (21.1%). This is lower than that reported in one local study conducted among patients with diabetes (46.2%) [73]. Compared to the studies that used the MEDAS tool, the prevalence is also lower than that in regional studies conducted in countries adopting Levantine cuisine, such as Lebanon and Jordan [74, 75], and in countries in the northern Mediterranean [76]. The low prevalence in the present study may be attributed to the population characteristics of young university students, who might be more inclined to adopt a Western-style diet than older populations. Indeed, adults older than 45 years in Lebanon demonstrated greater adherence to the MD than younger participants [75]. Some components of the MD, such as legumes, fruits, and vegetables, might be unappealing for young adults, especially since globalization has encouraged an inclination toward less optimal diets and lifestyles [77–80].

In the present study, no association was found between acne and the consumption of individual MD components, except for vegetables. The impact of fruits and vegetables on acne presentation has been controversial in the literature. While certain studies have suggested that fruits and vegetables may be protective against acne, a similar number of studies have reported no association [81–85]. Regardless of significance, the majority of these studies lacked the longitudinal design necessary for establishing a causal relationship. Additionally, no associations were found between acne and the intake of fish [83, 84], nuts [83], red meat [83, 84, 86], or sweetened drinks [84, 87, 88].

Moreover, only family history was associated with acne in this study. This finding aligns with the vast majority of studies, which have consistently reported an association between family history and acne presentation. Heng and Chew conducted a meta-analysis and reported a pooled odds ratio of 2.91, suggesting that acne presentation is associated with parental family history [51]. The genetic contribution to acne susceptibility has been investigated in multiple studies. Acne hereditability in twin studies has been estimated at approximately 80% [81, 89, 90]. Additionally, a total of 46 genomic loci associated with acne risk have been reported in molecular genetic studies [91–93].

This study was the first to investigate the impact of the MD on a dermatological disease among a non-European population. This study also contributes to the scarce literature investigating the impact of adherence to the MD on acne. However, this study has several limitations. First, retrospective, case‒control studies do not guarantee a valid temporal sequence of events. The retrospective nature weakens the attribution of acne as an outcome to adherence to the MD as an exposure, as the diet effect is accumulative and the exact onset of acne cannot be ascertained. Second, the exposure data may be inaccurate, as the tool used to assess dietary exposure depends on long-term memory. Moreover, the assessment of adherence to the MD was not performed by a dietician. Primary data collection through face-to-face interviews by well-trained people is essential to ensure accurate data collection and guide the target population. Furthermore, the identification of potential confounders was limited by the lack of consensus on the factors affecting acne and the inherent impracticality of accounting for every dietary factor in diet research. Finally, information bias may have been introduced, as BMI was calculated based on self-reported weight and height.

## Conclusions

Acne has been linked to Western-style diets with a high GI and low dairy consumption. Research has suggested that adherence to the MD, as a dietary system with low GI and high antioxidant properties, may have a protective role against acne. This study aimed to explore the effect of adherence to the MD on acne incidence. Neither adherence to the MD nor adherence to any of its components was found to be associated with acne diagnosis or severity. Those with acne were more likely to have a positive 1st -degree family history of acne. More prospective, longitudinal research into the impact of adherence to the MD on acne is recommended, especially in light of the scarcity of related studies and the variations in the methodologies and settings thereof. This is in addition to the methodological limitations in confounding and measurement inherent in diet research in general. Comprehensive, detailed and culturally specific definitions of the MD should be made available for use to guide research methodology. Additionally, cultural-specific references of food portions based on actual intake should be made available to further enhance data collection tools.

## Data Availability

The data collected and analyzed for this study are available from the corresponding author upon reasonable request.

## Abbreviations

aOR: Adjusted odds ratio
BMI: Body mass index
CI: Confidence interval
CVD: Cardiovascular disease
GAGS: Global Acne Grading System
GBD: Global Burden of Disease Study
GI: Glycemic index
IGF-1: Insulin-like growth factor-1
IRB: Institutional Review Board
MD: Mediterranean diet
MEDAS: Mediterranean Diet Adherence Screener
OR: Odds ratio
PCOS: Polycystic ovarian syndrome
WHO: World Health Organization

## References

[1] Institute for Health Metrics and Evaluation. Global Burden of Disease Study 2019 (GBD 2019) Data Resources. 2020. https://ghdx.healthdata.org/gbd-2019 (accessed February 28 2024).

[2] Institute for Health Metrics and Evaluation. Acne vulgaris — Level 3 cause. 2019. https://www.healthdata.org/results/gbd_summaries/2019/acne-vulgaris-level-3-cause (accessed February 29 2024).

[3] Chen H, Zhang TC, Yin XL, Man JY, Yang XR, Lu M (2022). Magnitude and temporal trend of acne vulgaris burden in 204 countries and territories from 1990 to 2019: an analysis from the global burden of Disease Study 2019. Br J Dermatol.

[4] Jaber RM, Alnshash BM, Mousa SN, Fayoumi HS, Al-Qaderi LM, Zant AM (2020). The epidemiology of acne vulgaris among adolescents and young adults in Jordan University Hospital. Open J Nurs.

[5] Dabash D, Salahat H, Awawdeh S, Hamadani F, Khraim H, Koni AA, Zyoud SH (2024). Prevalence of acne and its impact on quality of life and practices regarding self-treatment among medical students. Sci Rep.

[6] Al-Kubaisy W, Abdullah NN, Kahn SM, Zia M (2014). Sociodemographic characteristics of Acne among University students in Damascus, Syria. Epidemiol Res Int.

[7] Darwish MA, Al-Rubaya AA (2013). Knowledge, beliefs, and Psychosocial Effect of Acne Vulgaris among Saudi Acne patients. ISRN Dermatol.

[8] Stathakis V, Kilkenny M, Marks R (1997). Descriptive epidemiology of acne vulgaris in the community. Australas J Dermatol.

[9] Shen Y, Wang T, Zhou C, Wang X, Ding X, Tian S, Liu Y, Peng G, Xue S, Zhou J (2012). Prevalence of acne vulgaris in Chinese adolescents and adults: a community-based study of 17,345 subjects in six cities. Acta Derm Venereol.

[10] Collier CN, Harper JC, Cafardi JA, Cantrell WC, Wang W, Foster KW, Elewski BE (2008). The prevalence of acne in adults 20 years and older. J Am Acad Dermatol.

[11] Vos T, Flaxman AD, Naghavi M, Lozano R, Michaud C, Ezzati M, Shibuya K, Salomon JA, Abdalla S, Aboyans V (2012). Years lived with disability (YLDs) for 1160 sequelae of 289 diseases and injuries 1990–2010: a systematic analysis for the global burden of Disease Study 2010. Lancet.

[12] Melnik B (2012). Dietary intervention in acne: attenuation of increased mTORC1 signaling promoted by Western diet. Dermatoendocrinol.

[13] Kircik LH (2016). Advances in the understanding of the Pathogenesis of Inflammatory Acne. J Drugs Dermatol.

[14] Smith TM, Gilliland K, Clawson GA, Thiboutot D (2008). IGF-1 induces SREBP-1 expression and lipogenesis in SEB-1 sebocytes via activation of the phosphoinositide 3-kinase/Akt pathway. J Invest Dermatol.

[15] Kucharska A, Szmurlo A, Sinska B (2016). Significance of diet in treated and untreated acne vulgaris. Postepy Dermatol Alergol.

[16] Smith RN, Mann NJ, Braue A, Makelainen H, Varigos GA (2007). A low-glycemic-load diet improves symptoms in acne vulgaris patients: a randomized controlled trial. Am J Clin Nutr.

[17] Caperton C, Block S, Viera M, Keri J, Berman B (2014). Double-blind, placebo-controlled study assessing the effect of chocolate consumption in subjects with a history of Acne Vulgaris. J Clin Aesthet Dermatol.

[18] Cerman AA, Aktas E, Altunay IK, Arici JE, Tulunay A, Ozturk FY (2016). Dietary glycemic factors, insulin resistance, and adiponectin levels in acne vulgaris. J Am Acad Dermatol.

[19] Burris J, Rietkerk W, Shikany JM, Woolf K (2017). Differences in Dietary Glycemic load and hormones in New York City adults with no and Moderate/Severe acne. J Acad Nutr Diet.

[20] Smith RN, Mann NJ, Braue A, Makelainen H, Varigos GA (2007). The effect of a high-protein, low glycemic-load diet versus a conventional, high glycemic-load diet on biochemical parameters associated with acne vulgaris: a randomized, investigator-masked, controlled trial. J Am Acad Dermatol.

[21] Adebamowo CA, Spiegelman D, Danby FW, Frazier AL, Willett WC, Holmes MD (2005). High school dietary dairy intake and teenage acne. J Am Acad Dermatol.

[22] Adebamowo CA, Spiegelman D, Berkey CS, Danby FW, Rockett HH, Colditz GA, Willett WC, Holmes MD (2006). Milk consumption and acne in adolescent girls. Dermatol Online J.

[23] Penso L, Touvier M, Deschasaux M, Szabo de Edelenyi F, Hercberg S, Ezzedine K, Sbidian E (2020). Association between Adult Acne and Dietary behaviors: findings from the NutriNet-Sante prospective cohort study. JAMA Dermatol.

[24] Silverberg NB (2012). Whey protein precipitating moderate to severe acne flares in 5 teenaged athletes. Cutis.

[25] Pontes Tde C, Fernandes Filho GM, Trindade Ade S, Sobral Filho JF (2013). Incidence of acne vulgaris in young adult users of protein-calorie supplements in the city of Joao Pessoa–PB. Bras Dermatol.

[26] Jung JY, Kwon HH, Hong JS, Yoon JY, Park MS, Jang MY, Suh DH (2014). Effect of dietary supplementation with omega-3 fatty acid and gamma-linolenic acid on acne vulgaris: a randomised, double-blind, controlled trial. Acta Derm Venereol.

[27] Trichopoulou A, Lagiou P (1997). Healthy traditional Mediterranean diet: an expression of culture, history, and lifestyle. Nutr Rev.

[28] Trichopoulou A, Martinez-Gonzalez MA, Tong TY, Forouhi NG, Khandelwal S, Prabhakaran D, Mozaffarian D, de Lorgeril M (2014). Definitions and potential health benefits of the Mediterranean diet: views from experts around the world. BMC Med.

[29] Dontas AS, Zerefos NS, Panagiotakos DB, Vlachou C, Valis DA (2007). Mediterranean diet and prevention of coronary heart disease in the elderly. Clin Interv Aging.

[30] Martinez-Gonzalez MA, de la Fuente-Arrillaga C, Nunez-Cordoba JM, Basterra-Gortari FJ, Beunza JJ, Vazquez Z, Benito S, Tortosa A, Bes-Rastrollo M (2008). Adherence to Mediterranean diet and risk of developing diabetes: prospective cohort study. BMJ.

[31] Kastorini CM, Milionis HJ, Esposito K, Giugliano D, Goudevenos JA, Panagiotakos DB (2011). The effect of Mediterranean diet on metabolic syndrome and its components: a meta-analysis of 50 studies and 534,906 individuals. J Am Coll Cardiol.

[32] Lourida I, Soni M, Thompson-Coon J, Purandare N, Lang IA, Ukoumunne OC, Llewellyn DJ (2013). Mediterranean diet, cognitive function, and dementia: a systematic review. Epidemiology.

[33] Barrea L, Balato N, Di Somma C, Macchia PE, Napolitano M, Savanelli MC, Esposito K, Colao A, Savastano S (2015). Nutrition and psoriasis: is there any association between the severity of the disease and adherence to the Mediterranean diet?. J Transl Med.

[34] Barrea L, Fabbrocini G, Annunziata G, Muscogiuri G, Donnarumma M, Marasca C, Colao A, Savastano S (2018). Role of Nutrition and Adherence to the Mediterranean Diet in the Multidisciplinary Approach of Hidradenitis Suppurativa: evaluation of Nutritional Status and its Association with Severity of Disease. Nutrients.

[35] Chen P, Yang Z, Fan Z, Wang B, Tang Y, Xiao Y, Chen X, Luo D, Xiao S, Li J (2023). Associations of adherence to Mediterranean-Like diet pattern with incident rosacea: a prospective cohort study of government employees in China. Front Nutr.

[36] Schwingshackl L, Morze J, Hoffmann G (2020). Mediterranean diet and health status: active ingredients and pharmacological mechanisms. Br J Pharmacol.

[37] Cordain L, Lindeberg S, Hurtado M, Hill K, Eaton SB, Brand-Miller J (2002). Acne vulgaris: a disease of western civilization. Arch Dermatol.

[38] Kalaiselvan I, Samuthirapandi M, Govindaraju A, Sheeja Malar D, Kasi PD (2016). Olive oil and its phenolic compounds (hydroxytyrosol and tyrosol) ameliorated TCDD-induced heptotoxicity in rats via inhibition of oxidative stress and apoptosis. Pharm Biol.

[39] Skroza N, Tolino E, Semyonov L, Proietti I, Bernardini N, Nicolucci F, La Viola G, Del Prete G, Saulle R, Potenza C (2012). Mediterranean diet and familial dysmetabolism as factors influencing the development of acne. Scand J Public Health.

[40] Barrea L, Donnarumma M, Cacciapuoti S, Muscogiuri G, De Gregorio L, Blasio C, Savastano S, Colao A, Fabbrocini G (2021). Phase angle and Mediterranean diet in patients with acne: two easy tools for assessing the clinical severity of disease. J Transl Med.

[41] Ah-Thiane L, Nguyen JM, Khammari A, Dréno B (2022). Lifestyle habits and impact of the Mediterranean diet on facial acne severity in French women: a case-control study. Int J Womens Dermatol.

[42] Bertolani M, Rodighiero E, Saleri R, Pedrazzi G, Bertoli S, Leone A, Feliciani C, Lotti T, Satolli F (2022). The influence of Mediterranean diet in acne pathogenesis and the correlation with insulin-like growth factor-1 serum levels: implications and results. Dermatol Rep.

[43] Christiane Dabdoub N. Classic Palestinian cookery. Saqi Books; 2000.

[44] The Institute for Middle East Understanding (IMEU). Palestinian Cuisine | IMEU. 2006 2006. https://imeu.org/article/palestinian-cuisine (accessed September 29 2023).

[45] Keri JE. Acne Vulgaris - Dermatologic Disorders. 2022/02//. 2022. https://www.msdmanuals.com/professional/dermatologic-disorders/acne-and-related-disorders/acne-vulgaris (accessed October 25 2023).

[46] Thomas DC, Greenland S (1983). The relative efficiencies of matched and independent sample designs for case-control studies. J Chronic Dis.

[47] Geneva WCO, Organization WH (2000). Obesity: preventing and managing the global epidemic : report of a WHO consultation.

[48] Kelsey JL, Whittemore AS, Evans AS, Thompson, Douglas W. Methods in Observational Epidemiology, Second Edition, Second Edition edn. Oxford, New York: Oxford University Press; 1996.

[49] Hajian-Tilaki K (2011). Sample size estimation in epidemiologic studies. Casp J Intern Med.

[50] Hamdan M, Badrasawi M, Zidan S (2020). Factors Associated with adherence to the Mediterranean Diet among Palestinian High School females’ students in Hebron city: cross-sectional study. Iraq Med J.

[51] Heng AHS, Chew FT (2020). Systematic review of the epidemiology of acne vulgaris. Sci Rep.

[52] Schröder H, Fitó M, Estruch R, Martínez-González MA, Corella D, Salas-Salvadó J, Lamuela-Raventós R, Ros E, Salaverría I, Fiol M (2011). A short screener is valid for assessing Mediterranean diet adherence among older Spanish men and women. J Nutr.

[53] Martinez-Gonzalez MA, Garcia-Arellano A, Toledo E, Salas-Salvado J, Buil-Cosiales P, Corella D, Covas MI, Schroder H, Aros F, Gomez-Gracia E (2012). A 14-item Mediterranean diet assessment tool and obesity indexes among high-risk subjects: the PREDIMED trial. PLoS ONE.

[54] Estruch R, Ros E, Salas-Salvadó J, Covas MI, Corella D, Arós F, Gómez-Gracia E, Ruiz-Gutiérrez V, Fiol M, Lapetra J (2018). Primary Prevention of Cardiovascular Disease with a Mediterranean Diet supplemented with Extra-virgin Olive oil or nuts. N Engl J Med.

[55] Martinez-Gonzalez MA, Corella D, Salas-Salvado J, Ros E, Covas MI, Fiol M, Warnberg J, Aros F, Ruiz-Gutierrez V, Lamuela-Raventos RM (2012). Cohort profile: design and methods of the PREDIMED study. Int J Epidemiol.

[56] Estruch R, Ros E, Salas-Salvadó J, Covas MI, Corella D, Arós F, Gómez-Gracia E, Ruiz-Gutiérrez V, Fiol M, Lapetra J (2013). Primary prevention of cardiovascular disease with a Mediterranean diet. N Engl J Med.

[57] Hebestreit K, Yahiaoui-Doktor M, Engel C, Vetter W, Siniatchkin M, Erickson N, Halle M, Kiechle M, Bischoff SC (2017). Validation of the German version of the Mediterranean Diet Adherence Screener (MEDAS) questionnaire. BMC Cancer.

[58] Bekar C, Goktas Z (2023). Validation of the 14-item mediterranean diet adherence screener. Clin Nutr ESPEN.

[59] Mahdavi-Roshan M, Salari A, Soltanipour S (2018). Reliability and validity of the 14-point mediterranean diet adherence screener among the Iranian high risk population. Mediterr J Nutr Metab.

[60] Vallverdu-Queralt A, de Alvarenga JF, Estruch R, Lamuela-Raventos RM (2013). Bioactive compounds present in the Mediterranean sofrito. Food Chem.

[61] Doshi A, Zaheer A, Stiller MJ (1997). A comparison of current acne grading systems and proposal of a novel system. Int J Dermatol.

[62] Breslow NE, Day NE (1980). Statistical methods in Cancer Research volume I: the analysis of case-control studies.

[63] Ratner B (2010). Variable selection methods in regression: ignorable problem, outing notable solution. J Target Meas Anal Mark.

[64] Chowdhury MZI, Turin TC (2020). Variable selection strategies and its importance in clinical prediction modelling. Fam Med Community Health.

[65] World Medical Association (2013). World Medical Association Declaration of Helsinki: ethical principles for medical research involving human subjects. JAMA.

[66] Reiling J (2019). Hasty generalizations. JAMA.

[67] Triposkiadis F, Boudoulas KD, Xanthopoulos A, Boudoulas H (2021). Fallacies in medical practice: renin-angiotensin-aldosterone system inhibition and COVID-19 as a paradigm. Hellenic J Cardiol.

[68] Willett W (1987). Nutritional epidemiology: issues and challenges. Int J Epidemiol.

[69] Tarasuk VS, Brooker AS (1997). Interpreting epidemiologic studies of diet-disease relationships. J Nutr.

[70] Davis C, Bryan J, Hodgson J, Murphy K (2015). Definition of the Mediterranean Diet: A literature review. Nutrients.

[71] Badrasawi M, Altamimi M, Zidan S, Illner AK, Aleksandrova K (2022). Development and validation of a photographic food atlas of Middle Eastern Mediterranean diet: toward improved understanding of traditional healthy and sustainable diets. Front Nutr.

[72] Noah A, Truswell AS (2001). There are many Mediterranean diets. Asia Pac J Clin Nutr.

[73] Badrasawi M, Hamdan M, Al Tamimi M (2021). Quality of life and adherence to mediterranean diet among type 2 diabetes mellitus patients of a primary health care clinic in Hebron city, Palestine. Med J Nutr Metab.

[74] Zaidalkilani AT, Alhaj OA, Serag El-Dine MF, Fekih-Romdhane F, AlRasheed MM, Jahrami HA, Bragazzi NL (2021). Arab women adherence to the Mediterranean Diet and Insomnia. Med (Kaunas).

[75] Karam J, Ghach W, Bouteen C, Makary M-J, Riman M, Serhan M (2022). Adherence to Mediterranean diet among adults during the COVID-19 outbreak and the economic crisis in Lebanon. Nutr Food Sci.

[76] Obeid CA, Gubbels JS, Jaalouk D, Kremers SPJ, Oenema A (2022). Adherence to the Mediterranean diet among adults in Mediterranean countries: a systematic literature review. Eur J Nutr.

[77] Bhattacharya S, Juyal R, Hossain MM, Singh A (2020). Non-communicable diseases viewed as collateral damage of our decisions: fixing accountabilities and finding sloutions in primary care settings. J Family Med Prim Care.

[78] Goryakin Y, Lobstein T, James WP, Suhrcke M (2015). The impact of economic, political and social globalization on overweight and obesity in the 56 low and middle income countries. Soc Sci Med.

[79] Kopp W (2019). How western Diet and Lifestyle Drive the pandemic of obesity and civilization diseases. Diabetes Metab Syndr Obes.

[80] Rakhra V, Galappaththy SL, Bulchandani S, Cabandugama PK (2020). Obesity and the Western Diet: how we got Here. Mo Med.

[81] Wei B, Pang Y, Zhu H, Qu L, Xiao T, Wei HC, Chen HD, He CD (2010). The epidemiology of adolescent acne in North East China. J Eur Acad Dermatol Venereol.

[82] Aksu AE, Metintas S, Saracoglu ZN, Gurel G, Sabuncu I, Arikan I, Kalyoncu C (2012). Acne: prevalence and relationship with dietary habits in Eskisehir, Turkey. J Eur Acad Dermatol Venereol.

[83] Ismail NH, Manaf ZA, Azizan NZ (2012). High glycemic load diet, milk and ice cream consumption are related to acne vulgaris in Malaysian young adults: a case control study. BMC Dermatol.

[84] Karciauskiene J, Valiukeviciene S, Gollnick H, Stang A (2014). The prevalence and risk factors of adolescent acne among schoolchildren in Lithuania: a cross-sectional study. J Eur Acad Dermatol Venereol.

[85] Al Hussein S, Al Hussein H, Vari C-E, Todoran N, Al Hussein H, Ciurba A, Dogaru M. Diet, Smoking and Family History as potential risk factors in Acne Vulgaris – a community-based study. Acta Med Marisiensis 2016, 62.

[86] Di Landro A, Cazzaniga S, Cusano F, Bonci A, Carla C, Musumeci ML, Patrizi A, Bettoli V, Pezzarossa E, Caproni M (2016). Adult female acne and associated risk factors: results of a multicenter case-control study in Italy. J Am Acad Dermatol.

[87] Wolkenstein P, Misery L, Amici JM, Maghia R, Branchoux S, Cazeau C, Voisard JJ, Taieb C (2015). Smoking and dietary factors associated with moderate-to-severe acne in French adolescents and young adults: results of a survey using a representative sample. Dermatology.

[88] Suppiah TSS, Sundram TKM, Tan ESS, Lee CK, Bustami NA, Tan CK (2018). Acne Vulgaris and its association with dietary intake: a Malaysian perspective. Asia Pac J Clin Nutr.

[89] Bataille V, Snieder H, MacGregor AJ, Sasieni P, Spector TD (2002). The influence of genetics and environmental factors in the pathogenesis of acne: a twin study of acne in women. J Invest Dermatol.

[90] Evans DM, Kirk KM, Nyholt DR, Novac C, Martin NG (2005). Teenage acne is influenced by genetic factors. Br J Dermatol.

[91] Navarini AA, Simpson MA, Weale M, Knight J, Carlavan I, Reiniche P, Burden DA, Layton A, Bataille V, Allen M (2014). Genome-wide association study identifies three novel susceptibility loci for severe acne vulgaris. Nat Commun.

[92] Petridis C, Navarini AA, Dand N, Saklatvala J, Baudry D, Duckworth M, Allen MH, Curtis CJ, Lee SH, Burden AD (2018). Genome-wide meta-analysis implicates mediators of hair follicle development and morphogenesis in risk for severe acne. Nat Commun.

[93] Mitchell BL, Saklatvala JR, Dand N, Hagenbeek FA, Li X, Min JL, Thomas L, Bartels M, Jan Hottenga J, Lupton MK (2022). Genome-wide association meta-analysis identifies 29 new acne susceptibility loci. Nat Commun.

